# Immediate reconstruction of mandibular defect after treatment of medication-related osteonecrosis of the jaw (MRONJ) with rhBMP-2/ACS and miniplate: Review of 3 cases

**DOI:** 10.1016/j.ijscr.2019.11.038

**Published:** 2019-11-27

**Authors:** Mu-seong Kim, Kyung-jin Kim, Bok-joo Kim, Chul-hoon Kim, Jung-han Kim

**Affiliations:** Department of Oral and Maxillofacial Surgery, Dong-A University Medical Center, 26, Daesingongwon-ro Seo-gu Busan, Republic of Korea

**Keywords:** Medication-related osteonecrosis of the jaws, Bone morphogenic protein, Collagen sponge, Miniplate, Case series

## Abstract

**Introduction:**

The purpose of this study was to pursue, and to report the results of, mandibular reconstruction and rehabilitation of medication-related osteonecrosis of the jaw (MRONJ) patients having large critical-sized defects of the mandible using a combination of recombinant human bone morphogenetic protein-2 (rhBMP-2) and absorbable collagen sponge (ACS) with surgical miniplate without any grafting materials.

**Case presentation:**

Three (3) patients aged 67 and 86 (2 patients) presented due to discomfort on the mandible. They all had a medical history of bisphosphonate and steroids treatment orally or intravenously, and all had been diagnosed as MRONJ stage 3. Sequestrectomy and saucerization were performed, and then a surgical miniplate (Hansolmedical, Korea) was adapted and fixed on the sound portion of the mandible. rhBMP-2 was loaded onto an ACS at a dose of 1.5 mg/cc. Several rhBMP-2 (Cowellmedi, Korea)/ACS (Ateloplug, TRMkorea, Korea) were placed into the bony defect with a surgical miniplate. All 3 patients recovered without complications. They all exhibited radiographic evidence of bone formation by 3 months postoperatively in every case.

**Conclusions:**

All 3 patients were treated successfully with rhBMP-2/ACS and miniplate without any complications. This protocol reported herein represents a new approach to the surgical treatment of maxillofacial bone defects and deficiencies, especially in MRONJ patients.

## Highlights

• The pathogenesis of MRONJ is related to repression of osteoclast mediated bone remodeling.
• rhBMP-2 can stimulate not only osteoblasts but also osteoclasts and induce new bone formation.
• ACS has been proved to be a good carrier of rhBMP-2 with maximal efficacy.
• Application of rhBMP-2/ACS can be a new approach to surgical treatment for MRONJ patients.

## 1 Introduction

Bisphosphonates have been increasingly prescribed for patients with osteoporosis, Paget disease, hypercalcemia of malignancy, osteolytic-bone metastases, and multiple myeloma. Despite the many benefits of these drugs, osteonecrosis of the jaw (ONJ) is a significant complication [1]. A recent paper from the American Association of Oral and Maxillofacial Surgeons (AAOMS) suggests that bisphosphonate-related osteonecrosis of the jaw (BRONJ) should be renamed medication-related osteonecrosis of the jaw (MRONJ). This is because ONJ is turned out to be related with other anti-resorptive or anti-angiogenic medications including bisphosphonates. MRONJ is defined as an area of exposed bone in the maxillofacial region, or bone that can be probed through a fistula, which has persisted for more than 8 weeks in a patient who is currently taking or has previously taken anti-resorptive or anti-angiogenic medication and has had no history of radiotherapy. Although tooth extraction was performed in most initial reported case of ONJ, these teeth commonly had existing periodontal or periapical disease [2,3].

MRONJ can be categorized with staging system from stage 0 to stage 3 according to severity. Stage 2 is characterized by exposed and necrotic bone associated with infection as evidenced by pain & erythema, and stage 3 is defined as exposed necrotic bone extending beyond the region of alveolar bone [3]. Large defects on the mandible secondary to necrotic bone (generally MRONJ stage 2 or 3), if not appropriately treated, result in significant morbidity or pathologic fracture. Various bone-grafting and bone-manipulation techniques are available for restoration of large mandibular bony defects. Autogenous graft has been the “Gold Standard”; however, all of the commonly employed grafting procedures have limitations, including inconvenience and morbidity associated with harvesting of autogenous bone grafts. Indeed, the ideal technique for mandibular reconstruction has yet to be established [4].

The development of effective reconstruction procedures using growth factors without the need for conventional bone grafting have been reported in connection with reconstructive surgery of the head and neck area. Clinical use of growth factor can be divided into two main ways, autogenous growth factors from platelet concentrates (PCs) and human recombinant growth factors. The use of recombinant growth factors is currently focused on platelet derived growth factor (PDGF) and bone morphogenetic proteins (BMPs) [5]. BMPs are a group of osteo-inductive, sequentially arranged amino acids and polypeptides that are capable of stimulating stem cells to become osteoblastic cells and activate bone formation. Application of recombinant human bone morphogenetic protein-2 (rhBMP-2) as a substitute for necrotic-bone removal can be considered to be an effective and safe therapeutic option for reconstruction of localized large bone defects of the jaw [4].

The purpose of this study was to pursue, and to report the results of, the reconstruction and rehabilitation of 3 MRONJ patients having large critical-sized defects of the mandible using recombinant human bone morphogenetic protein-2 (rhBMP-2) with absorbable collagen sponge (ACS) as the retentive carrier and a surgical miniplate for stability without any grafting materials. This paper has been reported in line with the PROCESS criteria [6].

## 2 Case presentation

### 2.1 Patients and pre-surgical evaluation

We retrospectively evaluated 3 patients who had been treated at the Department of Oral and Maxillofacial Surgery for discomfort on the mandible. They all had a medical history of treatment with bisphosphonates and steroids orally or intravenously. The patients’ pre-surgical evaluation data is presented in Table 1.

**Table 1:** Pre-surgical evaluation of 3 cases.

	Case 1	Case 2	Case 3
Age/Gender	67/F	86/F	86/F
Location	Mandible Right	Mandible Anterior	Mandible Right
Disease	Osteoporosis IHP* HT**	Osteoporosis DM*** HT	Osteoporosis Pancreatic cancer Dementia HT
Medication	Oral BP**** and steroids	IV BP	Oral BP
Period	l0 years	5 years	8 years
Etiology	Periodontitis	Extraction	Periodontitis

IHP* : Idiopathic Hypertrophic Pachymeningitis.

HT** : Hypertension.

DM*** : Diabetes Mellitus.

BP**** : Bisphosphonate.

### 2.2 Surgical technique and post-surgical evaluation

**Fig. 1:**
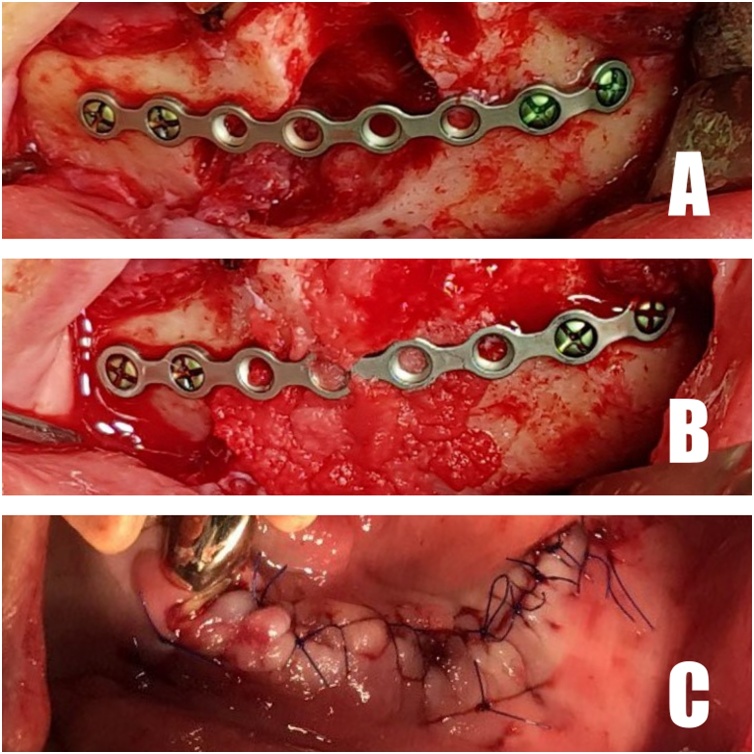
A. Fixation of miniplate. B. rhBMP-2/ACS placed. C. Soft-tissue closure.

Although three patients have different oral and medical history, they all had large defects on mandible and had been diagnosed as MRONJ stage 3. Surgery under general anesthesia was planned as a same surgical technique. The patients were well informed, and the surgical procedure was performed according to an intra-oral approach. The osteolytic lesions were exposed and extraction was done (if needed), after which, sequestrectomy and saucerization were performed. A surgical miniplate (Hansolmedical, Korea) was adapted and fixed on the sound portion of the mandible (Fig. 1A).

Subsequently, human recombinant bone morphogenetic protein-2 (rhBMP-2) was loaded onto an ACS at a dose of 1.5 mg/cc. Combinations of rhBMP-2 (CowellBMP, Cowellmedi, Korea) and ACS (Ateloplug, TRMkorea, Korea) were placed and filled fully into the bony defect with a surgical miniplate (Fig. 1B). The soft tissue was closed by tension-free closure (Fig. 1C).

The patients were re-assessed at various times and intervals based on panoramic radiography and/or cone-beam computed tomography (CBCT).

### 2.3 Case report 1

**Fig. 2:**
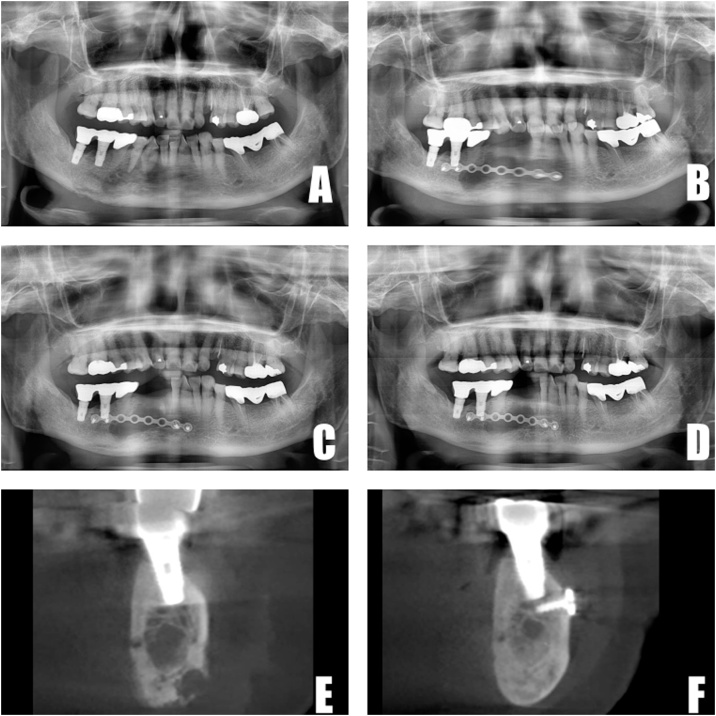
A. Panoramic X-ray at first visit. B. Panoramic X-ray 1 month postoperatively. C. Panoramic X-ray 3 months postoperatively. D. Panoramic X-ray 6 months postoperatively. E. CBCT at first visit. F. CBCT 6 months postoperatively.

A 67-year-old female presented with acute onset of pain on the mandibular right side. She had a medical history of osteoporosis and idiopathic hypertrophic pachymeningitis (IHP), and had taken bisphosphonates and steroids orally for 10 years. Three (3) months later (after a drug holiday and antibiotic therapy), surgery was performed. Under general anesthesia, the flap was elevated and anterior teeth (#42,43,44) were extracted. After saucerization and curettage, an 8-hole miniplate was fixed, and the defects were filled fully with rhBMP-2/ACS. The flap was then closed, and soft-tissue healing proceeded. Post-operative panoramic radiography was taken at 1-month, 3-month and 6-month intervals. Bone regeneration was observed comparing the first visit with 3 and 6 months postoperatively by both panoramic X-ray and CBCT (Fig. 2).

### 2.4 Case report 2

An 86-year-old female presented with swelling and a fistula on the anterior mandible after extraction of anterior mandibular teeth. She also had a history of osteoporosis, and had taken bisphosphonates intravenously every 3 months for 5 years. Two (2) months later (after antibiotics therapy), surgery was performed. Radiography was taken 3, 6 and 12 months postoperatively. The patient recovered well and soft-tissue healing was good at 2 weeks post-operatively. CBCT had been taken at the first visit and then at 6 months postoperatively. At 6 months postoperatively, plate removal was performed due to the patient’s request. At that time too, new-bone formation and pseudo periosteum were checked.

**Fig. 3:**
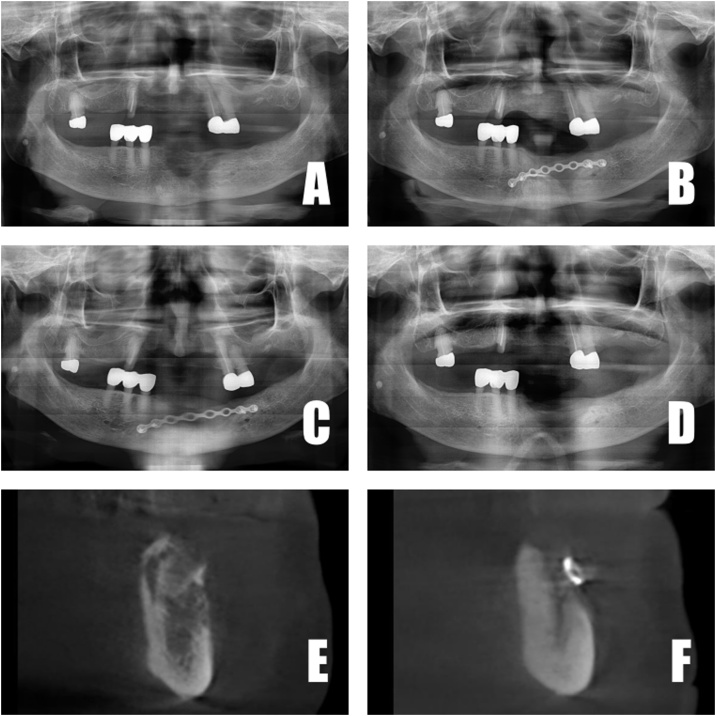
A. Panoramic X-ray at first visit. B. Panoramic X-ray 3 months postoperatively. C. Panoramic X-ray 6 months postoperatively. D. Panoramic X-ray 12 months postoperatively. E. CBCT at first visit. F. CBCT 6 months postoperatively.

Currently, the patient wears a removable partial denture prosthesis with no discomfort. A series of panoramic radiography and CBCT taken between the first visit and post-operatively also clearly showed bone regeneration (Fig. 3).

### 2.5 Case report 3

**Fig. 4:**
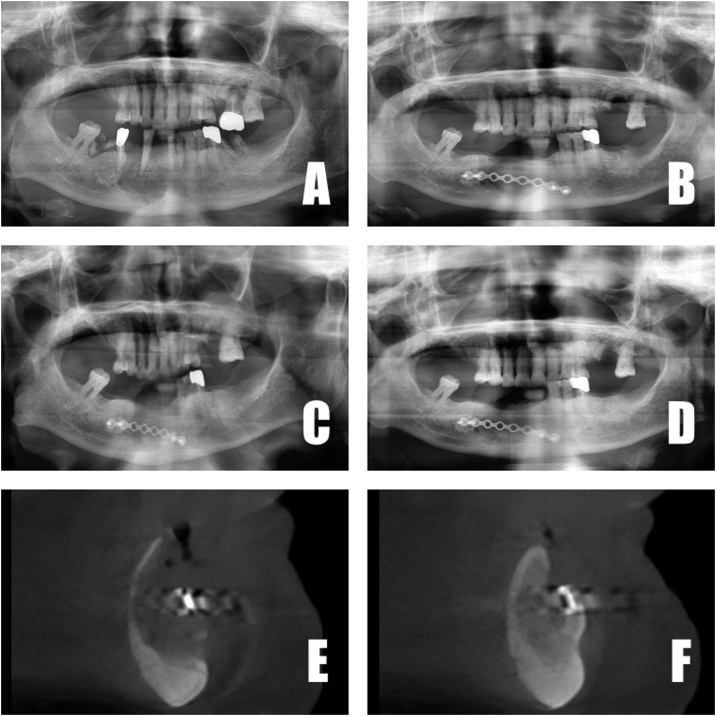
A. Panoramic X-ray at first visit. B. Panoramic X-ray 3 months postoperatively. E. Panoramic X-ray 6 months postoperatively. F. Panoramic X-ray 12 months postoperatively. G. CBCT after surgery. H. CBCT 6 months postoperatively.

An 86-year-old female presented for evaluation of the right mandible. The patient’s medical history was significant for osteoporosis and pancreatic cancer, and she also had an 8-year history of oral bisphosphonate medication. Without any drug holiday, surgery under general anesthesia was planned and proceeded as usual. After extraction of several teeth (#26,31,32,36,43,45,46), procedures were performed as per our established surgical protocol. At 3, 6 and 12 months postoperatively, panoramic radiography was taken, as was CBCT (both immediate-postoperatively and 6 months postoperatively). Both panoramic radiography and CBCT clearly showed new-bone formation (Fig. 4).

## 3 Discussion

All 3 patients exhibited radiographic evidence of bone formation at 3 months postoperatively (Fig. 2, Fig. 3, Fig. 4). Soft-tissue healing was good, and no complications arose. Recurrence at the 1-year follow up likewise had not occurred. In case 3, comparison of 1 month and 6 month post-operative CBCT revealed dramatic bone formation (Fig. 4E, F). Mandibular discomfort had subsided, and prosthetic rehabilitation was achieved at 6 months or 12 months postoperatively for all 3 patients. In other words, bone regeneration as well as soft-tissue healing was good in all 3 patients.

The treatment of MRONJ lesions is widely debated in terms of the timing of surgery, medical therapy and drug holiday. Surgical treatment ranges from conservative debridement of necrotic bone to major resection of the necrotic lesion. For several decades, the “Gold Standard” in the surgical management of large bone defects in MRONJ stage 2 or 3 has been autograft, which involves harvesting of healthy bone from one anatomical site. This technique bears considerable risks (donor-site pain, morbidity, infection, cost, and time). Especially, most MRONJ patients are old-aged, have many systemic diseases and are susceptible to infection or other complications. Thus, more conservative and safe techniques are needed for effective treatment of MRONJ patients.

Bone morphogenetic proteins (BMPs) have been well studied as stimulators of osteoblast differentiation and bone formation [7]. However, bone-marrow-derived cultures treated with recombinant human BMP-2 have shown increased RANKL-mediated osteoclastogenesis [9]. These results suggest that BMPs, acting on osteoclasts, play a larger role than previously perceived in regulating osteoclast differentiation and potentially in the bone-remodeling process as well [[7], [8], [9]].

The pathogenesis of MRONJ is related to bisphosphonate repression of osteoclast-mediated bone remodeling through disruption of intracellular pathways and inhibition of angiogenesis [10]. Thus, application of rhBMP-2 to MRONJ patients is considered reasonable treatment, especially as rhBMP-2 can stimulate not only osteoblasts but also osteoclasts. Moreover, rhBMP-2 without any graft materials can be employed as a more conservative therapy for MRONJ patients for whom no severe complications are anticipated.

The osteogenic effects of rhBMP-2 require combination with a biomaterial matrix to attain maximal efficacy. This should provide for retention of the protein for a sufficient period of time to affect the bone repair. The combination of rhBMP-2 with an ACS matrix has proven to be a very promising therapy in a variety of applications [11]. However, the collagen carrier lacks structural stability, and as such, might be compressed by soft-tissue walls. To avoid such occurrence, it requires the use of mechanical protectors such as titatnuim mesh, biocompatible membrane or surgical plate. For space maintenance and stability, a surgical miniplate was used in our surgical technique. Miniplate is highly used in maxillofacial surgery area and easy to handle. We can expect miniplate to prevent soft tissue collapse and prolong the initial releasing time of rhBMP-2. However, miniplate as well as other mechanical protectors could not prevent soft tissue migration perfectly and secondary surgery for removal of protectors might result in MRONJ again. Above all, primary closure is the most important procedure and it is technique-sensitive. For responsible application of rhBMP-2 to MRONJ patients, additional research in the development of ideal carrier with enough stability and releasing efficiency should be required.

## 4 Conclusions

All 3 patients were treated successfully with rhBMP-2/ACS and miniplate without any complications. This concept is representative of a new approach to the surgical treatment of maxillofacial bone defects and deficiencies, especially in MRONJ patients.

## Funding

This study received no specific grant from any funding agency.

## Ethical approval

This study was exempted from ethnical approval by Dong-A University Medical Center.

## Consent

Written informed consent was obtained from the patients for publication of these case reports.

## Author contribution

1) Pf. Jung-han Kim: the main surgeon; formulated surgery protocols
2) Dr. Mu-seong Kim: the 2nd main surgeon; drafted manuscript
3) Dr. Kyung-jin Kim: 1st assistant surgeon; collected data
4) Pf. Chul-hoon Kim: the supervisor; reviewed manuscript
5) Pf. Bok-joo Kim: the 2nd supervisor; reviewed manuscript

## Registration of research studies

UIN is researchregistry5147 at http://www.researchregistry.com.

## Guarantor

Chul-hoon Kim.

Bok-joo Kim.

## Provenance and peer review

Not commissioned, externally peer-reviewed.

## Declaration of Competing Interest

This research did not receive any specific grant from any funding agencies in the public, commercial, or not-for-profit sectors.
